# Volumetric abnormalities of thalamic subnuclei in medication-overuse headache

**DOI:** 10.1186/s10194-017-0791-5

**Published:** 2017-08-14

**Authors:** Zhiye Chen, Zhihua Jia, Xiaoyan Chen, Mengqi Liu, Shuangfeng Liu, Lin Ma, Shengyuan Yu

**Affiliations:** 1 Department of Radiology, Chinese PLA General Hospital, 28 Fuxing Road, Beijing, 100853 China; 2 Department of Neurology, Chinese PLA General Hospital, 28 Fuxing Road, Beijing, 100853 China; 3 Department of Radiology, Hainan Branch of Chinese PLA General Hospital, Sanya, 572013 China

**Keywords:** Medication-overuse headache, Migraine, Thalamus, Magnetic resonance imaging

## Abstract

**Background:**

The thalamus exerts a pivotal role in pain processing and cortical excitability control and a previous voxel-based morphometry study confirmed increased volume in bilateral thalamus in medication-overuse headache (MOH). The aim of this study is to investigate altered thalamic subnuclei volume in MOH compared with normal controls, and to evaluate the relationship of each thalamic subnuclei volume with the clinical variables.

**Methods:**

High resolution three-dimensional T1-weighted fast spoiled gradient recalled echo MR images were obtained from 27 patients with MOH and 27 normal controls (NC). Thalamic subnuclei templates were created based on Talairach template with MNI space transformation, and the individual thalamic subnuclei templates were generated by applying the deformation field from structural image segment to the thalamic subnuclei templates, and then individual thalamci subnuclei volume were calculated.

**Results:**

The whole thalamus and each thalamic subnuclei presented increased volume compared with NC (*P* < 0.05). The correlation analysis demonstrated that the whole thalamus volume and each thalamic subnuclei volume showed a negative relationship with HAMD scores(*P* < 0.05), and no any correlation with HAMA, VAS score and disease duration (*P* > 0.05).

**Conclusion:**

Increased gray matter volume in the whole thalamus and all the thalamus subnuclei may reflect central sensitization and higher-order of pain alteration in MOH. These structural changes in the thalamus may also be influenced by mood disturbances related to the MOH.

## Background

Medication-overuse headache (MOH) was defined as a headache occurring on 15 or more days per month developing as a consequence of regular overuse of acute or symptomatic headache medication for more than 3 months [1] . MOH has a prevalence of 0.6–2.0% in the general population [2, 3], and was associated with mood disorders in 27–85% and anxiety disorders in 61–83%. MOH patients experience reduced quality of life compared with those who do not suffer from headaches [4]. A pre-existing headache disorder seems to be required to develop MOH [5]. It is well known that previous primary headaches such as migraine are the most important risk factors for the development of MOH, 50%–70% MOH have co-occurrence of migraine in population-based studies [6, 7]. Many psychosocial and socioeconomic factors which are prevailed in patients with chronic forms of headache are also associated with MOH. However, the mechanism behind how chronic exposure to abortive medication leads to MOH remains unclear. Alteration of cortical neuronal excitability, central sensitization involving the trigeminal nociceptive system have been suggested to play a part in the pathophysiology of MOH [8].

The thalamus contains third-order trigeminovascular nociceptive neurons and exerts a pivotal role in pain processing and cortical excitability control [9, 10]. Microstructural and functional alterations of the thalamus have been found in migraine patients [11, 12]. Significant volume reductions of the following thalamic nuclei densely connected to the limbic system were observed in migraineurs: central nuclear complex, anterior nucleus and lateral dorsal nucleus, supported that higher-order integration systems are altered in migraine [11]. Increased iron deposition and myelin content/cellularity in the thalamus of migraine with aura patients compared with migraine without aura patients and healthy controls were found, may underlie abnormal cortical excitability control leading to cortical spreading depression and visual aura [13]. A voxel-based morphometry (VBM) study identified increased gray matter volume in bilateral thalamus in MOH patients [14]. Although it was demonstrated that periaqueductal gray (PAG) volume gain [15] and altered intrinsic functional connectivity architecture [16] were confirmed in MOH patients in our previous study, however, it was not known that how the thalamic subfields volume changed in MOH up to now.

Up to now, several documents had recognized that thalamic subnuclei were segmented based on diffusion tensor imaging [17, 18], and thalamic nuclei densely connected to the limbic system were observed in migraineurs [11]. Therefore, morphology analysis of thalamic subnuclei would provide more information in the understanding of neuromechanism of MOH.

The main objective of the current study was to investigate the altered thalamic subnuclei volume in MOH compared with normal controls, and to further evaluate the relationship of each thalamic subnuclei volume with the clinical variables.

## Methods

### Subjects

This study was approved by the ethics committee of the Chinese PLA General Hospital, and written informed consent was obtained from the subjects according to the Declaration of Helsinki. Twenty-seven MOH patients were consecutively recruited from the headache center, Chinese PLA General Hospital. The included criteria of MOH included as follows: (1) All patients with both, MOH and migraine; (2) The diagnosis of 8.2 MOH, 1.1 and 1.2 migraine based on the International Classification of Headache Disorders, third Edition (beta version) (ICHD-III beta); (3) Without migraine preventive medication in the past 3 months. The excluded criteria included as follows: (1) With chronic disorders, including hypertension, diabetes mellitus, cardiovascular diseases, etc.; (2) With cranium trauma, psychotic disorder, and regular use of a psychoactive or hormone medication. Part of MOH patients were overlapping with our previous studies [15, 16]. Twenty-seven normal controls (NCs) were recruited, who should never have any primary headache disorders or other types of headache in the past year, and had the same exclusion criteria with MOH patients. Headache information were registered and evaluated in our headache database. All the patients were given with the Visual Analogue Scale (VAS) evaluation. Additionally, we used the Hamilton Anxiety Scale (HAMA) [19] scale to assess the anxiety, the Hamilton Depression Scale) [20] to assess the depression, and the Mini-mental State Examination (MMSE) [21] to assess the cognitive function of all the participants. MRI scans were taken in the interictal stage at least three days after a migraine attack for MOH patients. Alcohol, nicotine, caffeine, and other substances were avoided for at least 12 h before MRI examination.

### MRI acquisition

MRI data were obtained by a conventional eight-channel quadrature head coil from a GE 3.0 T MR system (DISCOVERY MR750, GE Healthcare, Milwaukee, WI, USA). All subjects were instructed to lie in a supine position, and formed padding was used to limit head movement. An axial three-dimensional T1-weighted fast spoiled gradient recalled echo (3D T1-FSPGR) sequence was performed to acquire the brain structure images. The 3D T1-FSPGR parameters were listed as follows: TR (repetition time) = 6.3 ms, TE (echo time) = 2.8 ms, flip angle = 15o, FOV (field of view) = 25.6 cm × 25.6 cm, Matrix = 256 × 256, NEX (number of acquisition) = 1]. All the subjects were performed the same imaging protocols, and the subjects with structural damage would be excluded.

### Image processing

**Fig. 1:**
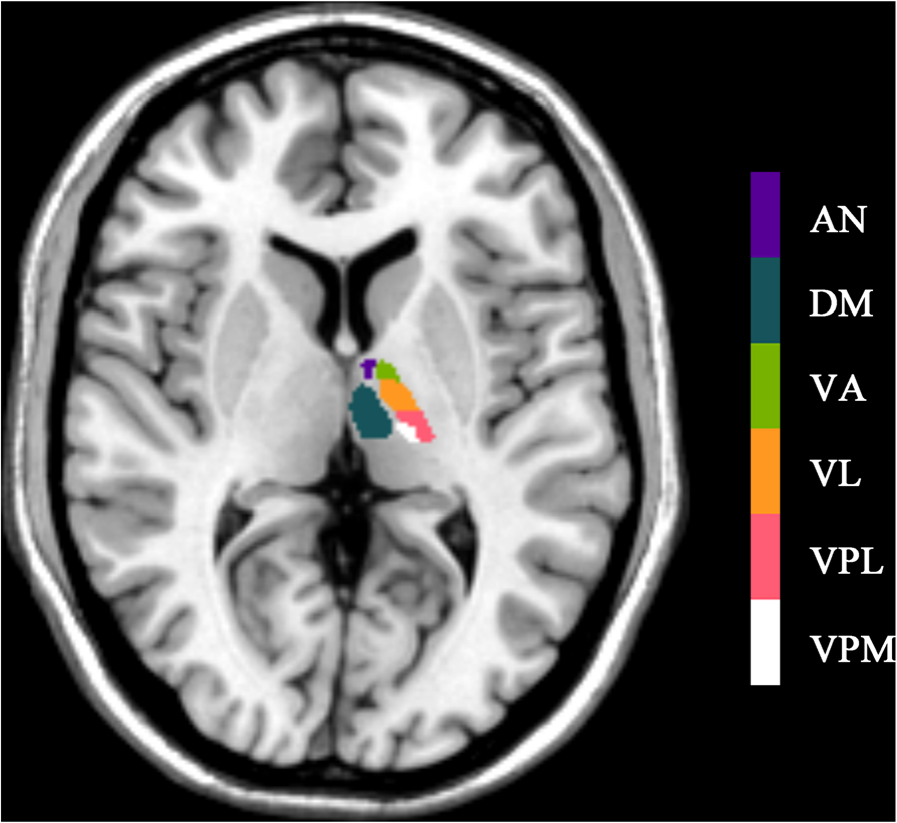
The standard thalamic subnuclei templates were created according to Talairach template. AN, anterior nucleus; DM, doromedial nucleus; VA, ventral anterior nucleus; VL, ventral lateral nucleus; VPL, ventral posterior lateral nucleus; VPM, ventral posterior medial nucleus

**Fig. 2:**
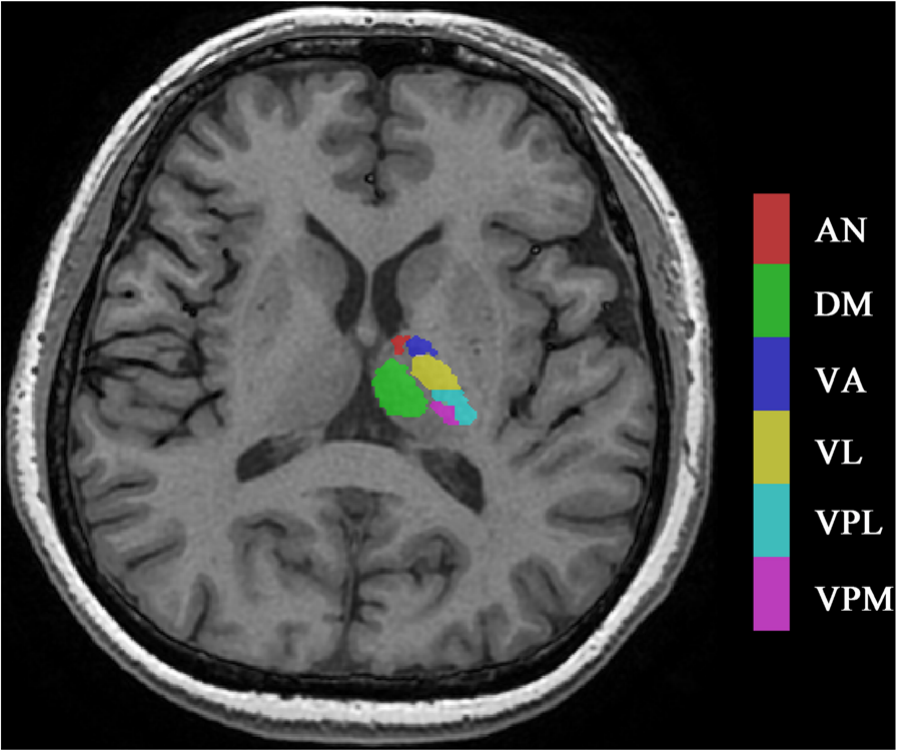
The individual thalamic nuclei were generated by applying the inverse deformation with pullback strategy to the standard thalamic subnuclei. AN, anterior nucleus; DM, doromedial nucleus; VA, ventral anterior nucleus; VL, ventral lateral nucleus; VPL, ventral posterior lateral nucleus; VPM, ventral posterior medial nucleus

Image processing mainly included the following steps: (1) Convert Talairach template [22] into MNI space, and the thalamic subnuclei templates were created using rest software [23]. The thalamic subnuclei [11] included following subregions: left/right ventral posterior lateral nucleus (L_VPL/R_VPL), left/right ventral posterior medial nucleus (L_VPM/R_VPM), left/right dorsomedial nucleus (L_DM/R_DM), left/right ventral lateral nucleus (L_VL/R_VL), left/right ventral anterior nucleus (L_VA/R_VA), and left/right anterior nucleus (L_AN/R_AN). (Fig. 1). (2) The individual structural images were segmented by the new segment tool embedded in SPM 12 software (http://www.fil.ion.ucl.ac.uk/spm), and the inverse deformation field was generated (iy_subjectID.nii). Then, the standard thalamic subnuclei were applied with the inverse deformation with pullback strategy, which would generate the individual thalamic subnuclei masks [15] (Fig. 2). Each individual thalamic subnucleus segmentations were visually inspected to confirm anatomical accuracy by one experienced radiologist. (3) The volume of individual thalamic subnuclei were measured by ITK-SNAP (V3.6.0) software (http://www.itksnap.org/pmwiki/pmwiki.php).

### Statistical analysis

The statistical analysis was performed by using PASW Statistics 18.0. The age, MMSE, HAMD,and HAMA were performed with independent samples T test, and sex was performed with Chi-Square test. The significance differences of whole thalamus and thalamic subnuclei volume were computed using analysis of covariance with the age and sex as covariates between MOH group and NC group. The Pearson’s correlation analysis was applied between thalamic volume and the clinical variables (including disease duration, VAS) in MOH. Significant difference was set at a *P* value of <0.05.

## Results

### Comparison of clinical characteristics between MOH and NC

The current study included 27 MOH patients (F/M = 20/7) and 27 normal controls (F/M = 19/8). The age, sex and MMSE showed no significant difference between MOH and NC (*P* > 0.05). There was a significant HAMD and HAMA between MOH (20.85 ± 12.67 and 17.70 ± 8.63) and NC (7.32 ± 4.26 and 9.78 ± 2.91) (*P* < 0.05)(Table 1).

**Table 1:** The clinical characteristics of the subjects

	MOH	NC	T value	*P* value
Num(F/M)	27(20/7)	27(19/8)	0.092^a^	0.761
Age	39.93 ± 9.75	43.04 ± 10.82	2.007	0.272
MMSE	27.41 ± 3.74	28.19 ± 1.00	2.007	0.302
HAMD	20.85 ± 12.67	7.32 ± 4.26	2.008	0.000
HAMA	17.70 ± 8.63	9.78 ± 2.91	2.007	0.000
DD	18.07 ± 9.85	NA	NA	NA
VAS	8.26 ± 1.46	NA	NA	NA

^a^Chi-square test. *MOH* medication-overuse headache, *NC* normal control, *DD* disease duration, *VAS* Visual Analogue Scale, *HAMA* Hamilton Anxiety Scale, *HAMD* Hamilton Depression Scale, *MMSE* Mini-mental State Examination, *NA* not available

### Comparison of thalamic subnuclei volume between MOH and NC

**Fig. 3:**
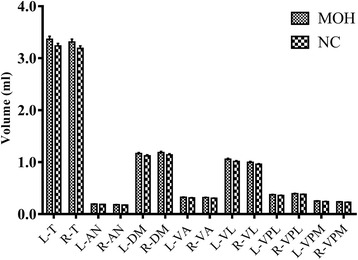
The mean volume of thalamic subnuclei in MOH and NC. L, left; R, right; AN, anterior nucleus; DM, doromedial nucleus; VA, ventral anterior nucleus; VL, ventral lateral nucleus; VPL, ventral posterior lateral nucleus; VPM, ventral posterior medial nucleus

Table 2 demonstrated that all the thalamic subnuclei presented increased volume in MOH compared with NC (*P* < 0.05). Bilateral whole thalamus also showed increased volume in MOH (L_T, 3.365 ± 0.291 ml, R_T,3.312 ± 0.288 ml) compared with NC (L_T, 3.237 ± 0.249 ml, R_T, 3.190 ± 0.241 ml) (*P* < 0.05) (Fig. 3).

**Table 2:** The volume comparison of thalamic subnuclei(ml) between MOH and NC

	MOH	NC	F value	*P* value
L_AN	0.195 ± 0.017	0.187 ± 0.015	6.788	0.012
L_DM	1.164 ± 0.106	1.123 ± 0.093	4.627	0.036
L_VA	0.324 ± 0.027	0.311 ± 0.023	6.974	0.011
L_VL	1.056 ± 0.090	1.014 ± 0.076	7.570	0.008
L_VPL	0.375 ± 0.033	0.360 ± 0.027	7.546	0.008
L_VPM	0.253 ± 0.022	0.243 ± 0.018	7.087	0.010
L_T	3.365 ± 0.291	3.237 ± 0.249	6.474	0.014
R_AN	0.182 ± 0.016	0.175 ± 0.013	6.657	0.013
R_DM	1.845 ± 0.112	1.143 ± 0.095	4.529	0.038
R_VA	0.319 ± 0.027	0.306 ± 0.022	8.365	0.006
R_VL	0.997 ± 0.083	0.958 ± 0.069	7.607	0.008
R_VPL	0.393 ± 0.034	0.379 ± 0.028	6.059	0.017
R_VPM	0.236 ± 0.021	0.229 ± 0.017	4.937	0.031
R_T	3.312 ± 0.288	3.190 ± 0.241	6.210	0.016

*L* left, *R* right, *AN* anterior nucleus, *DM* dorsomedial nucleus, *VA* ventral anterior nucleus, *VL* ventral lateral nucleus, *VPL* ventral posterior lateral nucleus, *VPM* ventral posterior medial nucleus, *T* thalamus

### Correlation analysis between thalamic subnuclei volume and the clinical variable

**Fig. 4:**
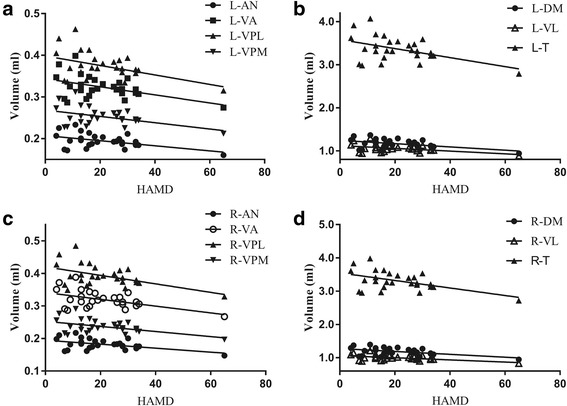
The scatter plot between thalamic subnuclei volume and the HAMD score (**a** and **b**, left thalamic subnuclei; **c** and **d**, right thalamic subnuclei). L, left; R, right; AN, anterior nucleus; DM, doromedial nucleus; VA, ventral anterior nucleus; VL, ventral lateral nucleus; VPL, ventral posterior lateral nucleus; VPM, ventral posterior medial nucleus; T, thalamus; HAMD, Hamilton Depression Scale

Figure 4 demonstrated that all the thalamic subnuclei volume were significantly negatively related with HAMD score (*P* < 0.05), and there were not significant relationship between all the thalamic subnuclei and the other clinical variables including HAMA, VAS and disease duration (*P* > 0.05) (Table 3).

**Table 3:** The correlation of thalamic subnuclei volume with the clinical variables

	HAMD		HAMA		VAS		DD	
	r	*P* value	r	*P* value	r	*P* value	r	*P* value
L_AN	−0.469	0.016	−0.159	0.439	0.002	0.991	−0.018	0.929
L_DM	−0.466	0.017	−0.171	0.404	0.025	0.902	0.071	0.724
L_VA	−0.467	0.016	−0.192	0.347	−0.073	0.718	−0.009	0.966
L_VL	−0.468	0.016	−0.186	0.363	−0.069	0.733	−0.005	0.981
L_VPL	−0.460	0.018	−0.178	0.384	−0.099	0.623	0.026	0.897
L_VPM	−0.453	0.020	−0.161	0.434	−0.087	0.668	0.035	0.863
L_T	−0.471	0.015	−0.180	0.380	−0.037	0.856	0.028	0.889
R_AN	−0.491	0.011	−0.165	0.420	0.027	0.892	−0.013	0.948
R_DM	−0.502	0.009	−0.205	0.316	0.014	0.946	0.077	0.701
R_VA	−0.512	0.008	−0.229	0.261	−0.058	0.775	0.018	0.931
R_VL	−0.514	0.007	−0.217	0.288	−0.064	0.753	0.037	0.853
R_VPL	−0.506	0.008	−0.215	0.292	−0.109	0.589	0.052	0.796
R_VPM	−0.494	0.010	−0.200	0.329	−0.108	0.592	0.055	0.786
R_T	−0.513	0.007	−0.213	0.297	−0.037	0.853	0.052	0.798

*L* left, *R* right, *AN* anterior nucleus, *DM* dorsomedial nucleus, *VA* ventral anterior nucleus, *VL* ventral lateral nucleus, *VPL* ventral posterior lateral nucleus, *VPM* ventral posterior medial nucleus, *T* thalamus

## Discussion

Our study aimed to identify morphological changes of thalamic subnuclei in MOH and try to reveal more information about the neuromechanism of MOH. In our study, psychiatric evaluation revealed that the majority of patients had comorbid psychiatric conditions, containing both anxiety and depressive disorders, which is accordant with epidemiologic studies [24, 25]. A previous study showed MOH patients have a greater risk of suffering from anxiety and depression than episodic migraine, and psychiatric disorders occurred significantly more often before the transformation from migraine into MOH than after [26]. It deduced that these disorders may be a risk factor for the evolution of migraine into MOH. Another follow-up study identified several risk factors for MOH among people with chronic headache, including increased Hospital Anxiety and Depression Scale score [27]. However, depression and anxiety disorders are associated with both migraine and non-migrainous headache, and this was related to the headache frequency rather than headache diagnosis in another research, so the relationship between psychiatric disorders and MOH may be comobidity [28]. The cause-effect relationship needs further longitudinal study.

Consistent with the previous study [14], we found increased whole thalamus volume bilaterally in the MOH. An increase in GMV may reflect structural brain plasticity as a result of exercise and learning [29]. Gray matter volume increase in the thalamus has also been found in chronic pain conditions such as back pain [30] and chronic post-traumatic headache [31]. Increased GMV in the thalamus might reflect central sensitization in chronic pain states. However, studies about the thalamic subnuclei volume in these chronic pain conditions have not been found. If the morphological abnormalities of thalamic subnuclei are specific to MOH or if the morphological abnormalities of thalamic can be normalized as cephalic, extra-cephalic pressure-pain thresholds and pain-related cortical potentials in MOH patients after withdrawal of the overused medication needs further study [31, 32]. Unlike the specific thalamic subnuclei decreases observed in migraineurs [11], all the thalamic subnuclei presented increased volume in MOH. Each thalamus is divided into the following subnuclei according to the inner medullary plate (including plate core): anterior nucleus (AN), dorsomedial nucleus (DM), ventral anterior nucleus (VA), ventral lateral nucleus (VL), ventral posterior lateral nucleus (VPL) and ventral posterior medial nucleus (VPM). AN of the thalamus is a key component of the hippocampal system for episodic memory. Via its connections with the anterior cingulate and orbitomedial prefrontal cortex, the AN may also involve in emotional and executive functions [33]. Affective and anxiety disorders prevailed in patients with chronic forms or transform of headache and substance use than in patients with migraine alone [34]. Decreased AN volume in migraineurs may be related to the psychiatric disorders in migraine patients and suggest that the central reorganization after repeated, long-term nociceptive signaling. Increased volume of AN in our study may suggest pre-existed morphological abnormalities in MOH. Somatosensory-related thalamic structures can be broadly divided into lateral and medial subdivisions (VPL and VPM), which receive sensory inputs from the spinal cord or medulla to the thalamus directly through the spinothalamic tract or trigeminothalamic tract [35]. VPL and VPM then project to the dorsal part of thalamus and then sends axon projections to the cerebral cortex for a complete sensory transmission [36]. Increased gray matter volume in DM, VPL and VPM may indicate higher-order of pain are altered in MOH.

In our study, we did not find a relation between the volumes of thalamic nuclei and clinical features, such as VAS or the duration of the disorder. It suggests that increased gray matter volume in thalamus may relate to the genetic background of patients with MOH. We observed negative associations between HAMD scores and gray matter volume in all the thalamus subnuclei in patients, suggesting that these structural changes may also be influenced by mood disturbances related to the disorder [37].

## Conclusions

In conclusion, increased gray matter volume in the whole thalamus and all the thalamus subnuclei may reflect central sensitization and higher-order of pain alteration in MOH. These structural changes in the thalamus may also be influenced by mood disturbances related to the MOH. Whether the observed morphological abnormalities in MOH can be reversed after withdrawal of the overused medication remains unclear.

## Abbreviations

AN: Anterior nucleus
DM: Dorsomedial nucleus
MOH: Medication-overuse headache
NC: Normal controls
VA: Ventral anterior nucleus
VL: Ventral lateral nucleus
VPL: Ventral posterior lateral nucleus
VPM: Ventral posterior medial nucleus
